# Ethyl *N*′-[(*E*)-4-hydroxy­benzyl­idene]hydrazinecarboxyl­ate at 123 K

**DOI:** 10.1107/S1600536808019818

**Published:** 2008-07-05

**Authors:** Xiang-Wei Cheng

**Affiliations:** a Zhejiang Police College Experience Center, Zhejiang Police College, Hangzhou 310053, People’s Republic of China

## Abstract

The mol­ecule of the title compound, C_10_H_12_N_2_O_3_, adopts a *trans* configuration with respect to the C=N bond. The dihedral angle between the benzene ring and the hydrazinecarboxyl­ate plane is 14.6 (1)°. Mol­ecules are linked into a three-dimensional network by O—H⋯O, N—H⋯O and C—H⋯O hydrogen bonds, and by C—H⋯π inter­actions.

## Related literature

For general background, see: Parashar *et al.* (1988 ▶); Hadjoudis *et al.* (1987 ▶); Borg *et al.* (1999 ▶). For a related structure, see: Shang *et al.* (2007 ▶).
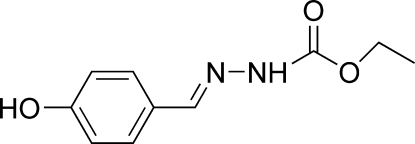


## Experimental

### 

#### Crystal data

C_10_H_12_N_2_O_3_
*M* *_r_* = 208.22
Orthorhombic, 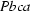

*a* = 11.342 (3) Å
*b* = 7.6114 (17) Å
*c* = 24.986 (5) Å
*V* = 2157.0 (9) Å^3^
*Z* = 8
Mo *K*α radiation
μ = 0.10 mm^−1^
*T* = 123 (2) K
0.26 × 0.25 × 0.23 mm

#### Data collection

Bruker SMART CCD area-detector diffractometer
Absorption correction: multi-scan (*SADABS*; Bruker, 2002 ▶) *T*_min_ = 0.965, *T*_max_ = 0.968
21084 measured reflections
1900 independent reflections
1521 reflections with *I* > 2σ(*I*)
*R*_int_ = 0.040

#### Refinement

*R*[*F* ^2^ > 2σ(*F* ^2^)] = 0.040
*wR*(*F* ^2^) = 0.125
*S* = 1.02
1900 reflections
137 parameters
H-atom parameters constrained
Δρ_max_ = 0.21 e Å^−3^
Δρ_min_ = −0.18 e Å^−3^

### 

Data collection: *SMART* (Bruker, 2002 ▶); cell refinement: *SAINT* (Bruker, 2002 ▶); data reduction: *SAINT*; program(s) used to solve structure: *SHELXS97* (Sheldrick, 2008 ▶); program(s) used to refine structure: *SHELXL97* (Sheldrick, 2008 ▶); molecular graphics: *SHELXTL* (Sheldrick, 2008 ▶); software used to prepare material for publication: *SHELXTL*.

## Supplementary Material

Crystal structure: contains datablocks I, global. DOI: 10.1107/S1600536808019818/ci2620sup1.cif

Structure factors: contains datablocks I. DOI: 10.1107/S1600536808019818/ci2620Isup2.hkl

Additional supplementary materials:  crystallographic information; 3D view; checkCIF report

## Figures and Tables

**Table 1:** Hydrogen-bond geometry (Å, °) *Cg*1 is the centroid of the C1–C6 ring.

*D*—H⋯*A*	*D*—H	H⋯*A*	*D*⋯*A*	*D*—H⋯*A*
O1—H1⋯O2^i^	0.82	1.96	2.752 (2)	161
N2—H2*A*⋯O2^ii^	0.86	2.11	2.936 (2)	161
C9—H9*B*⋯O1^iii^	0.97	2.57	3.425 (3)	148
C2—H2⋯*Cg*1^iv^	0.93	2.97	3.636 (2)	130
C5—H5⋯*Cg*1^v^	0.93	2.77	3.613 (2)	151

Symmetry codes: (i) 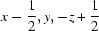; (ii) 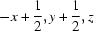; (iii) 
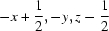
; (iv) 
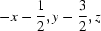
; (v) 
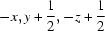
.

**Fig. 1.**
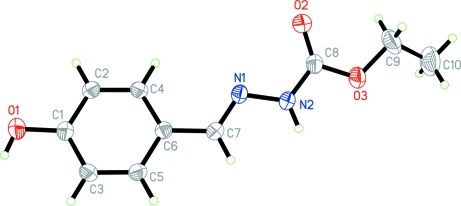
Molecular structure of the title compound, showing 30% probability displacement ellipsoids and the atomic numbering.

**Fig. 2.**
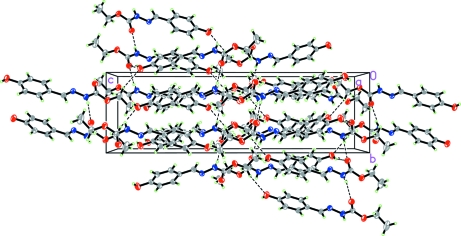
Crystal packing of the title compound, viewed approximately down the a axis. Dashed lines indicate intermolecular hydrogen bonds.

## supplementary crystallographic information

### Comment

Benzaldehydehydrazone derivatives have received considerable attention for a long time due to their pharmacological activity (Parashar *et al.*, 1988) and their photochromic properties (Hadjoudis *et al.*, 1987). They are important intermidiates for 1,3,4-oxadiazoles, which have been reported to be versatile compounds with many properties (Borg *et al.*, 1999). As a further investigation of this type of derivatives, the crystal structure of the title compound is reported here.

The title molecule (Fig.1) adopts a trans configuration with respect to the C═N bond. The hydrazine carboxylic acid ethyl ester group is slightly twisted away from the attached ring. The dihedral angle between the C1–C6 ring and the C7/C8/N1/N2/O2/O3 plane is 14.6 (1)°. The bond lengths and angles agree with those observed for N'-(4-methoxybenzylidene)methoxyformohydrazide (Shang *et al.*, 2007).

In the crystal structure, O—H···O, N—H···O and C—H···O hydrogen bonds and C—H···π interactions (Table 1) link the molecules into a three-dimensional network (Fig.2).

### Experimental

4-Hydroxybenzaldehyde (12.2 g, 0.1 mol) and ethyl hydrazinecarboxylate (10.4 g, 0.1 mol) were dissolved in methanol (50 ml) with stirring and left for 6 h at room temperature. The resulting solid was filtered off and recrystallized from ethanol to give the title compound in 90% yield. Single crystals suitable for X-ray analysis were obtained by slow evaporation of an ethanol solution at room temperature (m.p. 460–462 K).

### Refinement

H atoms were positioned geometrically (N—H = 0.86 Å, O—H = 0.82 Å and C—H = 0.93–0.97 Å) and refined using a riding model, with *U*_iso_(H) = 1.2*U*_eq_(C,N) and 1.5*U*_eq_(O).

### Crystal data

**Table:**

C_10_H_12_N_2_O_3_	*F*_000_ = 880
*M**_r_* = 208.22	*D*_x_ = 1.282 Mg m^−^^3^
Orthorhombic, *P**b**c**a*	Mo *K*α radiation λ = 0.71073 Å
Hall symbol: -P 2ac 2ab	Cell parameters from 1900 reflections
*a* = 11.342 (3) Å	θ = 1.6–25.0º
*b* = 7.6114 (17) Å	µ = 0.10 mm^−^^1^
*c* = 24.986 (5) Å	*T* = 123 (2) K
*V* = 2157.0 (9) Å^3^	Block, colourless
*Z* = 8	0.26 × 0.25 × 0.23 mm

### Data collection

**Table:**

Bruker SMART CCD area-detector diffractometer	1900 independent reflections
Radiation source: fine-focus sealed tube	1521 reflections with *I* > 2σ(*I*)
Monochromator: graphite	*R*_int_ = 0.040
*T* = 123(2) K	θ_max_ = 25.0º
φ and ω scans	θ_min_ = 1.6º
Absorption correction: multi-scan(SADABS; Bruker, 2002)	*h* = −13→13
*T*_min_ = 0.965, *T*_max_ = 0.968	*k* = −8→9
21084 measured reflections	*l* = −28→29

### Refinement

**Table:**

Refinement on *F*^2^	Hydrogen site location: inferred from neighbouring sites
Least-squares matrix: full	H-atom parameters constrained
*R*[*F*^2^ > 2σ(*F*^2^)] = 0.040	*w* = 1/[σ^2^(*F*_o_^2^) + (0.0722*P*)^2^ + 0.4536*P*] where *P* = (*F*_o_^2^ + 2*F*_c_^2^)/3
*wR*(*F*^2^) = 0.125	(Δ/σ)_max_ = 0.001
*S* = 1.02	Δρ_max_ = 0.21 e Å^−^^3^
1900 reflections	Δρ_min_ = −0.18 e Å^−^^3^
137 parameters	Extinction correction: SHELXL97 (Sheldrick, 2008), Fc^*^=kFc[1+0.001xFc^2^λ^3^/sin(2θ)]^-1/4^
Primary atom site location: structure-invariant direct methods	Extinction coefficient: 0.0110 (18)
Secondary atom site location: difference Fourier map	

### Special details

**Table:**

Geometry. All e.s.d.'s (except the e.s.d. in the dihedral angle between two l.s. planes) are estimated using the full covariance matrix. The cell e.s.d.'s are taken into account individually in the estimation of e.s.d.'s in distances, angles and torsion angles; correlations between e.s.d.'s in cell parameters are only used when they are defined by crystal symmetry. An approximate (isotropic) treatment of cell e.s.d.'s is used for estimating e.s.d.'s involving l.s. planes.
Refinement. Refinement of *F*^2^ against ALL reflections. The weighted *R*-factor *wR* and goodness of fit *S* are based on *F*^2^, conventional *R*-factors *R* are based on *F*, with *F* set to zero for negative *F*^2^. The threshold expression of *F*^2^ > σ(*F*^2^) is used only for calculating *R*-factors(gt) *etc*. and is not relevant to the choice of reflections for refinement. *R*-factors based on *F*^2^ are statistically about twice as large as those based on *F*, and *R*- factors based on ALL data will be even larger.

### Fractional atomic coordinates and isotropic or equivalent isotropic displacement parameters (Å^2^)

**Table:**

	*x*	*y*	*z*	*U*_iso_*/*U*_eq_	
O2	0.38975 (10)	0.12445 (15)	0.06297 (5)	0.0550 (4)	
O1	0.10016 (11)	0.07264 (18)	0.38449 (5)	0.0662 (4)	
H1	0.0396	0.1121	0.3978	0.099*	
O3	0.31948 (13)	0.34186 (17)	0.01045 (5)	0.0708 (4)	
N1	0.23119 (12)	0.22542 (18)	0.13972 (5)	0.0472 (4)	
N2	0.23619 (13)	0.29838 (19)	0.08895 (5)	0.0533 (4)	
H2A	0.1859	0.3769	0.0794	0.064*	
C4	0.21890 (13)	0.1206 (2)	0.25066 (6)	0.0451 (4)	
H4	0.2850	0.0839	0.2317	0.054*	
C6	0.13472 (13)	0.2248 (2)	0.22515 (6)	0.0422 (4)	
C1	0.10857 (14)	0.1271 (2)	0.33239 (6)	0.0459 (4)	
C3	0.02413 (14)	0.2324 (2)	0.30815 (7)	0.0493 (4)	
H3	−0.0410	0.2707	0.3275	0.059*	
C7	0.14800 (14)	0.2813 (2)	0.16958 (7)	0.0471 (4)	
H7	0.0940	0.3608	0.1555	0.056*	
C2	0.20628 (14)	0.0712 (2)	0.30315 (6)	0.0481 (4)	
H2	0.2629	0.0004	0.3192	0.058*	
C5	0.03729 (14)	0.2800 (2)	0.25500 (7)	0.0491 (4)	
H5	−0.0197	0.3501	0.2389	0.059*	
C8	0.32053 (15)	0.2449 (2)	0.05487 (6)	0.0489 (4)	
C9	0.3972 (3)	0.2864 (3)	−0.03288 (9)	0.1029 (9)	
H9A	0.4749	0.2601	−0.0188	0.123*	
H9B	0.3663	0.1809	−0.0495	0.123*	
C10	0.4055 (3)	0.4246 (4)	−0.07193 (10)	0.1096 (10)	
H10A	0.4564	0.3880	−0.1005	0.164*	
H10B	0.4372	0.5282	−0.0554	0.164*	
H10C	0.3285	0.4497	−0.0859	0.164*	

### Atomic displacement parameters (Å^2^)

**Table:**

	*U*^11^	*U*^22^	*U*^33^	*U*^12^	*U*^13^	*U*^23^
O2	0.0572 (7)	0.0535 (7)	0.0542 (7)	0.0039 (6)	0.0023 (5)	0.0014 (5)
O1	0.0693 (8)	0.0818 (9)	0.0475 (8)	0.0116 (7)	0.0088 (6)	0.0139 (6)
O3	0.1000 (10)	0.0651 (8)	0.0472 (7)	0.0138 (7)	0.0168 (7)	0.0131 (6)
N1	0.0533 (8)	0.0472 (8)	0.0412 (8)	0.0007 (6)	0.0011 (6)	0.0059 (6)
N2	0.0639 (9)	0.0534 (8)	0.0426 (8)	0.0116 (7)	0.0048 (7)	0.0112 (6)
C4	0.0428 (8)	0.0455 (9)	0.0469 (9)	0.0038 (7)	0.0050 (7)	−0.0010 (7)
C6	0.0431 (8)	0.0387 (8)	0.0447 (9)	−0.0018 (6)	−0.0001 (6)	0.0012 (6)
C1	0.0508 (9)	0.0447 (9)	0.0421 (9)	−0.0033 (7)	0.0001 (7)	0.0022 (7)
C3	0.0426 (8)	0.0528 (10)	0.0526 (10)	0.0030 (7)	0.0077 (7)	0.0009 (8)
C7	0.0495 (9)	0.0447 (9)	0.0471 (10)	0.0040 (7)	−0.0013 (7)	0.0053 (7)
C2	0.0484 (8)	0.0478 (9)	0.0483 (10)	0.0063 (7)	−0.0029 (7)	0.0024 (7)
C5	0.0437 (8)	0.0501 (9)	0.0534 (10)	0.0061 (7)	0.0001 (7)	0.0069 (7)
C8	0.0592 (10)	0.0455 (9)	0.0418 (9)	−0.0046 (8)	0.0000 (7)	0.0011 (7)
C9	0.155 (3)	0.0936 (18)	0.0598 (14)	0.0209 (17)	0.0467 (16)	0.0063 (12)
C10	0.107 (2)	0.155 (3)	0.0666 (15)	0.0021 (19)	0.0209 (13)	0.0293 (16)

### Geometric parameters (Å, °)

**Table:**

O2—C8	1.224 (2)	C1—C3	1.388 (2)
O1—C1	1.3695 (19)	C1—C2	1.394 (2)
O1—H1	0.82	C3—C5	1.385 (2)
O3—C8	1.3329 (19)	C3—H3	0.93
O3—C9	1.458 (3)	C7—H7	0.93
N1—C7	1.276 (2)	C2—H2	0.93
N1—N2	1.3860 (18)	C5—H5	0.93
N2—C8	1.344 (2)	C9—C10	1.437 (3)
N2—H2A	0.86	C9—H9A	0.97
C4—C2	1.372 (2)	C9—H9B	0.97
C4—C6	1.395 (2)	C10—H10A	0.96
C4—H4	0.93	C10—H10B	0.96
C6—C5	1.398 (2)	C10—H10C	0.96
C6—C7	1.462 (2)		
			
C1—O1—H1	109.5	C4—C2—C1	120.03 (14)
C8—O3—C9	116.92 (16)	C4—C2—H2	120.0
C7—N1—N2	115.58 (13)	C1—C2—H2	120.0
C8—N2—N1	119.19 (14)	C3—C5—C6	121.20 (15)
C8—N2—H2A	120.4	C3—C5—H5	119.4
N1—N2—H2A	120.4	C6—C5—H5	119.4
C2—C4—C6	121.41 (14)	O2—C8—O3	123.93 (15)
C2—C4—H4	119.3	O2—C8—N2	125.36 (15)
C6—C4—H4	119.3	O3—C8—N2	110.71 (15)
C4—C6—C5	117.91 (15)	C10—C9—O3	109.4 (2)
C4—C6—C7	122.06 (14)	C10—C9—H9A	109.8
C5—C6—C7	119.99 (14)	O3—C9—H9A	109.8
O1—C1—C3	122.78 (14)	C10—C9—H9B	109.8
O1—C1—C2	117.48 (14)	O3—C9—H9B	109.8
C3—C1—C2	119.74 (15)	H9A—C9—H9B	108.2
C5—C3—C1	119.70 (15)	C9—C10—H10A	109.5
C5—C3—H3	120.1	C9—C10—H10B	109.5
C1—C3—H3	120.1	H10A—C10—H10B	109.5
N1—C7—C6	122.24 (15)	C9—C10—H10C	109.5
N1—C7—H7	118.9	H10A—C10—H10C	109.5
C6—C7—H7	118.9	H10B—C10—H10C	109.5
			
C7—N1—N2—C8	−179.28 (15)	C3—C1—C2—C4	0.2 (2)
C2—C4—C6—C5	0.9 (2)	C1—C3—C5—C6	−0.3 (3)
C2—C4—C6—C7	178.80 (15)	C4—C6—C5—C3	−0.3 (2)
O1—C1—C3—C5	−178.91 (15)	C7—C6—C5—C3	−178.25 (15)
C2—C1—C3—C5	0.4 (2)	C9—O3—C8—O2	7.1 (3)
N2—N1—C7—C6	−176.50 (13)	C9—O3—C8—N2	−173.16 (19)
C4—C6—C7—N1	6.8 (3)	N1—N2—C8—O2	6.4 (3)
C5—C6—C7—N1	−175.38 (15)	N1—N2—C8—O3	−173.41 (14)
C6—C4—C2—C1	−0.9 (2)	C8—O3—C9—C10	−166.9 (2)
O1—C1—C2—C4	179.54 (14)		

### Hydrogen-bond geometry (Å, °)

**Table:**

*D*—H···*A*	*D*—H	H···*A*	*D*···*A*	*D*—H···*A*
O1—H1···O2^i^	0.82	1.96	2.752 (2)	161
N2—H2A···O2^ii^	0.86	2.11	2.936 (2)	161
C9—H9B···O1^iii^	0.97	2.57	3.425 (3)	148
C2—H2···Cg1^iv^	0.93	2.97	3.636 (2)	130
C5—H5···Cg1^v^	0.93	2.77	3.613 (2)	151

Symmetry codes: (i) *x*−1/2, *y*, −*z*+1/2; (ii) −*x*+1/2, *y*+1/2, *z*; (iii) −*x*+1/2, −*y*, *z*−1/2; (iv) −*x*−1/2, *y*−3/2, *z*; (v) −*x*, *y*+1/2, −*z*+1/2.
